# Mitochondrial-Targeted Molecular Imaging in Cardiac Disease

**DOI:** 10.1155/2017/5246853

**Published:** 2017-05-30

**Authors:** Jinhui Li, Jing Lu, You Zhou

**Affiliations:** ^1^Department of Chinese Medicine & Rehabilitation, Second Affiliated Hospital, Zhejiang University School of Medicine, Hangzhou 310009, China; ^2^Department of Nuclear Medicine, Second Affiliated Hospital, Zhejiang University School of Medicine, Hangzhou 310009, China; ^3^Zhejiang University Medical PET Center, Zhejiang University, Hangzhou 310009, China; ^4^Institute of Nuclear Medicine and Molecular Imaging, Zhejiang University, Hangzhou 310009, China; ^5^Key Laboratory of Medical Molecular Imaging of Zhejiang Province, Hangzhou 310009, China; ^6^Department of Neurobiology, Key Laboratory of Medical Neurobiology of Ministry of Health of China, Hangzhou, China; ^7^Zhejiang Province Key Laboratory of Mental Disorder's Management, Department of Psychiatry, First Affiliated Hospital, Zhejiang University School of Medicine, Hangzhou, China

## Abstract

The present study aimed to discuss the role of mitochondrion in cardiac function and disease. The mitochondrion plays a fundamental role in cellular processes ranging from metabolism to apoptosis. The mitochondrial-targeted molecular imaging could potentially illustrate changes in global and regional cardiac dysfunction. The collective changes that occur in mitochondrial-targeted molecular imaging probes have been widely explored and developed. As probes currently used in the preclinical setting still have a lot of shortcomings, the development of myocardial metabolic activity, viability, perfusion, and blood flow molecular imaging probes holds great potential for accurately evaluating the myocardial viability and functional reserve. The advantages of molecular imaging provide a perspective on investigating the mitochondrial function of the myocardium in vivo noninvasively and quantitatively. The molecular imaging tracers of single-photon emission computed tomography and positron emission tomography could give more detailed information on myocardial metabolism and restoration. In this study, series mitochondrial-targeted ^99m^Tc-, ^123^I-, and ^18^F-labeled tracers displayed broad applications because they could provide a direct link between mitochondrial dysfunction and cardiac disease.

## 1. Introduction

Therapies for coronary artery disease (CAD) and its related disorders have benefited from the developments in modern technology over the past few decades. Noninvasive examination of myocardial molecular imaging has proved to be of great diagnostic significance and has been widely accepted [1]. Recently, functional molecular imaging, such as single-photon emission computed tomography (SPECT) and positron emission tomography (PET), promises to expand the ability to evaluate cardiac diseases compared with the magnetic resonance imaging, ultrasound, and coronary angiography imaging modalities [2]. For instance, the assessment of myocardial function and perfusion imaging of heart ischemia using single SPECT is a widely used technique [3]. Myocardial molecular imaging using PET scanning is useful due to its predictive values; it assesses metabolic activity or viability through ^18^F-deoxyglucose (^18^F-FDG) in the living body [4]. Moreover, myocardial perfusion imaging (MPI) using SPECT is difficult to detect due to reduced myocardial blood flow (MBF) [5], which might be because of its low sensitivity. In contrast, PET imaging provides robust and sensitive measurements of MPI; it offers an excellent resolution, high sensitivity, lower tissue attenuation, and semiquantification or absolute quantification of MBF [6]. Many findings suggest using PET tracers because of their sensitivity and dynamic-imaging capabilities [7, 8] for myocardial function and viability detection [9, 10]. PET imaging is very complicated for nuclear cardiologists in contrast to SPECT perfusion imaging, which is still the most commonly used nuclear imaging technique in clinical practice. However, precise changes in myocardial metabolism, viability, and apoptosis that reflect mitochondrial activity at the molecular imaging level have not yet been systematically summarized.

**Figure 1:**
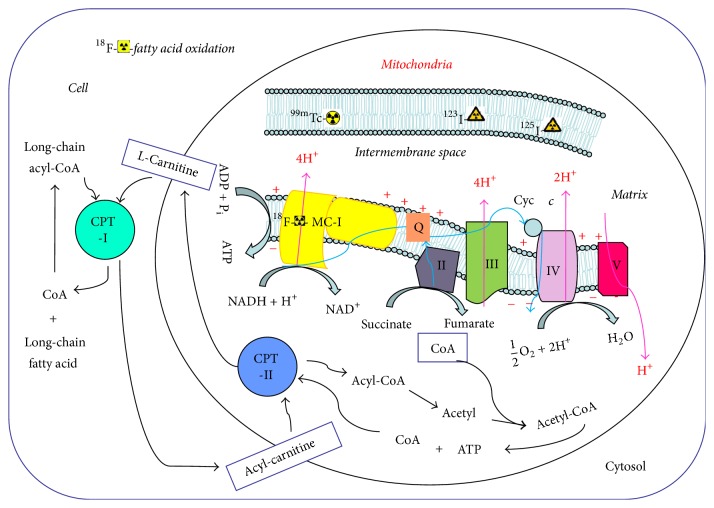
Chart patterns of complex composition, energy production, and fatty acid metabolism in mitochondria.

The mitochondrion comprises an outer membrane, an intermembrane space, an inner membrane, cristae, and matrix [11]. Physiologically, mitochondrial-rich heart tissue demands high energy in the form of adenosine triphosphate (ATP) and fatty acid all the time [12]. The inner membrane of mitochondria maintains a transmembrane gradient of ions and contains five complexes of essential membrane proteins, including nicotinamide adenine dinucleotide (NADH) dehydrogenase [also called mitochondrial complex I (MC-I)] [13]. The main function of MC-I is to transport NADH-reduced nicotinamide adenine along the respiratory electron transport chain, carried by proton transfer [14]. In this way, MC-I, MC-III, and MC-IV form a proton electrochemical gradient across the membrane, which is then used by MC-V to synthesize ATP [15]. Thus, inhibiting the activity of MC-I affects and reduces the synthesis of ATP (Figure 1). Moreover, MC-I inhibits the respiratory chain electron leakage resulting from the generation of reactive oxygen species, which induces oxidative damage to membrane lipids, proteins, and DNA [16]. Moreover, alterations in mitochondrial fatty acid oxidation (FAO) contribute to cardiac pathology. Therefore, mitochondrial dysfunction may reflect the dynamics of myocardial energy metabolism and apoptosis in some cardiac diseases such as myocardial infarction, chronic heart failure, and cardiomyopathy. No more existing molecular imaging data are available to describe MC-II, MC-III, MC-IV, and MC-V for cardiac diseases [17]. The outer mitochondrial membrane is thought to be the last barrier between the mitochondrion and the cytoplasm [18]. The permeability of outer membrane might regulate coupled cellular respiration and apoptosis [19].

This study intended to highlight the development of several mitochondrial-targeted SPECT and PET molecular imaging probes and to elucidate the advantages and disadvantages of molecular imaging for cardiac diseases. These probes would be presented to monitor and evaluate the changes in cardiac metabolic activity, viability, perfusion, and blood flow in preclinical and clinical practices (Table 1).

## 2. Mitochondrial-Targeted SPECT Agents

### 2.1. Mitochondrial Membrane as ^99m^Tc-Labeled SPECT Agents

Many ^99m^Tc-labeled mitochondrial-targeted imaging tracers have shown larger myocardial accumulations compared with other non-mitochondrial-targeted radiotracers. For instance, Galaris et al. reported that possibly cytochrome oxidase, localized in the mitochondrial inner membrane, is responsible for the binding of ^99m^Tc-gluconate [20]. ^99m^Tc-gluconate is mainly used for renal and myocardial scintigraphy. However, the ^99m^Tc-gluconate is not very stable after formulation the solution compared to ^99m^Tc-sestamibi and ^99m^Tc-tetrofosmin. Meanwhile, ^99m^Tc-gluconate imaging might get blur renal scintigraphy in patients with renal failure and dehydration [21]. Another classic radiotracer, ^99m^Tc-sestamibi (^99m^Tc-MIBI), is an isocyanate compound, with a low molecular weight suitable for SPECT imaging. ^99m^Tc-MIBI is a lipophilic cation imaging tracer, localized primarily in mitochondria [22]. It is a passive transport process, and the myocardial uptake of ^99m^Tc-MIBI is approximately 1.2%–1.5% of the injected dose, showing very low redistributive properties of the tracer compared with ^201^thallium (^201^TI). Most of the accumulated ^99m^Tc-MIBI is related to mitochondrial uptake. In contrast, part of the accumulated ^99m^Tc-tetrofosmin inside the rat myocardial cells enters the mitochondria [23]. Moreover, ^99m^Tc-MIBI perfusion imaging is considered useful for assessing mitochondrial function to monitor the severity of myocardial ischemia in patients with coronary artery stenosis [24, 25] or possible cardiotoxic drug evaluation.

Both ^99m^Tc-MIBI and ^99m^Tc-tetrofosmin are the most popular available radiotracers with widespread clinical applications all over the world and with virtually no biodistributive difference. Compared with ^201^Tl, ^99m^Tc-MIBI and ^99m^Tc-tetrofosmin afford better count statistics and image quality. However, ^99m^Tc-MIBI and ^99m^Tc-tetrofosmin are limited by low first-pass myocardial extraction [26], resulting in underestimation of the MBF at high flow rates, and have relatively high liver uptake [27], thus making it difficult to assess myocardial perfusion, particularly in the inferior left ventricular wall.

The mitochondrial membrane is the only permeable membrane for molecules with appropriate molecular charge and shape, while the mitochondrial potentials provide a driving force for the mitochondrial localization of ^99m^Tc tracers. The lipophilicity might modulate the penetration capability across mitochondrial membranes [28]. [^99m^Tc-N(mpo)(PNP5)]^+^ (^99m^Tc-N-MPO) and ^99m^Tc-[bis-(dimethoxypropylphosphinoethyl)-ethoxyethylamine-(PNP5)] [bis (N-ethoxyethyl)-dithiocarbamato (DBODC)] nitride (^99m^Tc-N-DBODC5) showed that liver clearance was fast, resulting in excellent heart/liver ratios, of which ^99m^Tc-N-MPO at 30 min postinjection was 12.75 ± 3.34, which was four times higher than that of ^99m^Tc-MIBI (2.90 ± 0.62) and two times higher than that of ^99m^Tc-DBODC5 (6.01 ± 1.45) in Sprague–Dawley rats [29, 30]. This indicated that ^99m^Tc-N-MPO has a lower first-pass extraction fraction compared with ^99m^Tc-MIBI. Moreover, planar imaging studies demonstrated that [^99m^Tc-(CO)_3_(15C5-PNP)]^+^ has a better liver clearance compared with ^99m^Tc-MIBI [31] and that [^99m^Tc-N(etma)(PNP5)]^+^ is a promising candidate for preclinical evaluations in rats [32]. These ^99m^Tc-based tracers, except for ^99m^Tc-DBODC5, allow SPECT images of the left ventricle and demonstrate favorable biodistribution in humans because of their high heart uptake and fast liver or lung washout features.

Recently, another subcellular distribution study showed that more than 73% of tri-methoxy-tris-pyrazolyl-^99m^Tc-(CO)_3_ (^99m^Tc-TMEOP) was associated with the mitochondrial fraction. No significant difference in mitochondrial accumulation was reported between the two tracers when compared with ^99m^Tc-MIBI [33, 34]. Overall, despite the fact that ^99m^Tc-MIBI has been used widely in the clinic, ^99m^Tc-tetrofosmin, ^99m^Tc-N-MPO, ^99m^Tc-15C5-PNP, ^99m^Tc-N-DBODC5, and ^99m^Tc-TMEOP have great potential compared with ^99m^Tc-MIBI [35]. ^99m^Tc-N-DBODC5 has better radiochemistry features of faster liver clearance and higher heart/organ count ratio compared with ^99m^Tc-MIBI both in stress or rest MPI in clinical trials [36].

### 2.2. MC-I Inhibitor as ^123^I-Labeled SPECT Agents

^123^I-labeled rotenone derivative (^123^I-CMICE-013) has been produced using a simple one-step synthetic procedure with an improved radiochemical yield and high stability compared with its uptake with tetrofosmin, sestamibi, and ^201^Tl in a porcine model of stress-induced myocardial ischemia [37]. In contrast, ^125^I-labeled tracers based on rotenone, which is a neutral and lipophilic MC-I inhibitor, involved multistep syntheses, resulting in a low yield of product [38]. The properties and biological characterization of ^123^I-CMICE-013 exhibited clear myocardium visualization with a low background activity in the lung and liver [39]. The ^123^I-CMICE-013 also showed promise in MPI, and the percentage injected dose of ^123^I-CMICE-013 taken up by the heart was greater than ^201^Tl, tetrofosmin, or sestamibi in humans.

7′-(Z)-^123^I-Iodorotenone (^123^I-ZIROT) has a good linear blood flow track over a wider range and other promising new MPI tracers [40]. ^123^I-ZIROT SPECT molecular imaging could improve diagnosis and show a better quantitative estimation of the severity of flow impairment for cardiovascular diseases. In spite of ^125^I-iodorotenone with a half-life of 60 days, the ability of ^123^I-labeled rotenone with a half-life of 13 h indicated that ^123^I-labeled iodorotenone could be used for SPECT perfusion imaging in clinical practices.

### 2.3. FAO as ^123^I-Labeled SPECT Agents

^123^I-labeled 15-(p-iodophenyl)-3-(R, S)-methyl pentadecanoic acid (BMIPP) is mainly trapped in the myocardium as triglycerides, depending on the ATP levels; 10%–20% of them are metabolized through *α*-oxidation after *β*-oxidation. Hirai et al. used dual-SPECT images of BMIPP and ^201^TI obtained 3 days and 24 days after the left coronary artery occlusion in the male Wistar-Kyoto rat model, respectively, to clarify the precise mechanism of the regulatory pathway of BMIPP in rats. The results demonstrated that BMIPP molecular imaging correlates well with the activities of 3-hydroxyacyl-coenzyme A dehydrogenase and citrate synthase, reflecting the deterioration of both fatty acid metabolism and citrate cycle [41]. Although the pharmacokinetics of ^123^I-BMIPP could not be used to evaluate FAO, it could detect an antecedent cardiac ischemic abnormality, such as angina pectoris and myocardial infarction. The main advantage of ^123^I-BMIPP was that the abnormal heart does not shift from fatty acids to glucose consumption for 0–24 h. Therefore, the defect of BMIPP does not normalize, whereas the perfusion defect can change instantaneously between the occurrence of heart failure and actual scanning time, since one has to consider the time to transport to the hospital, getting scanning done, and so on.

## 3. Mitochondrial-Targeted PET Agents

PET is the primary molecular imaging technique, with its high resolution and better sensitivity in vivo [42, 43]. Compared with other radiotracers, the ^18^F radiotracer could improve stability and bioavailability of tissue metabolism, enhance the bonding force, and reduce the plasma protein binding rate [44]. Incorporating ^18^F into a compound alters the lipophilic parameter and might increase the intrinsic activity, metabolic stability, and bioavailability [45]. Mitochondrial-targeted myocardial agents from the intake mechanism can be roughly divided into three categories: MC-I inhibitor derivatives [46], lipophilic cations [47–49], and FAO. In detail, the MC-I inhibitor analogue could bind specifically to the myocardial mitochondria; the lipophilic cation is used to enter the mitochondrial membrane potential; and FAO is a major pathway of energy production in the normal perfusion myocardium.

This study mainly addressed a large number of applications of myocardial molecular imaging of mitochondrial-targeted PET agents, such as analogues of MC-I inhibitors, ^18^F-labeled voltage sensor, and FAO tracer in preclinical or clinical trials.

### 3.1. MC-I Inhibitors as PET Agents

#### 3.1.1. ^18^F-Radiolabeled Rotenone Analogue

Rotenone analogue is a potential deposited MBF tracer. The myocardial extraction, retention, washout, and uptake were evaluated. Marshall found that the uptake of 7′-^18^F-fluoro-6′,7′-dihydrorotenone (^18^F-FDHR) increased compared with ^201^TI in the isolated rabbit heart [50]. Therefore, ^18^F-FDHR potentially provides better image resolutions compared with ^201^TI in other mammal models or healthy subjects.

#### 3.1.2. ^18^F-Flurpiridaz

^18^F-Flurpiridaz, also called ^18^F-BMS-747158-02, is a fluorine 18-labeled agent that binds to MC-I and is also designed to be a novel myocardial perfusion PET imaging agent. The clinical trial results of ^18^F-flurpiridaz PET imaging showed better evaluation of patients with known or suspected CAD having rapid uptake and slow washout properties. ^18^F-Flurpiridaz not only exhibited high and sustained cardiac uptake that was proportional to blood flow but also showed a clear and sustained cardiac uptake in rats, rabbits, and nonhuman primates with lower lung interference and rapid liver clearance compared with ^201^TI or ^99m^Tc-MIBI [51, 52]. In phase 1 clinical studies, the mean effective dose of ^18^F-flurpiridaz injected at rest was similar to that of ^18^F-FDG [53]. ^18^F-Flurpiridaz had an excellent image quality with two forms of drug-induced stress imaging and with the advantages of safety, favorable biodistribution, and excellent myocardial imaging characteristics. In the clinical phase 2 trial, PET MPI with ^18^F-flurpiridaz was shown to have an excellent sensitivity, a greater magnitude of reversible defects, a higher percentage of good images, and a higher diagnostic certainty of interpretation compared with SPECT. However, the specificity was not significantly different (PET 76.5% versus SPECT 73.5%) [54, 55]. To evaluate the myocardial function in comparison with ^13^N-ammonia [56], investigators concluded that, at least in a pig model, ^18^F-flurpiridaz used over a wide flow range was useful for clinical PET/computed tomography (CT) applications in the workup of subjects with suspected or proven CAD. This radiotracer has been enrolled in a phase 3 trial. Furthermore, taking advantage of the early kinetics, quantification of MBF in humans with ^18^F-flurpiridaz was feasible over a wide range of cardiac flow in the presence or absence of stress myocardial ischemia [57]. Lately, the PET probe 2-tert-butyl-4-chloro-5-2H-pyridazin-3-one (^18^F-BCPP-EF) showed a promising quantitative imaging of the MC-I activity. The ^18^F-BCPP-EF and ^18^F-flurpiridaz uptake was higher in the heart compared with the brain, muscle, and bone 60 min after the injection. Moreover, both ^18^F-BCPP-EF and ^18^F-flurpiridaz significantly decreased after preadministering rotenone in the heart [58].

#### 3.1.3. ^18^F-Fenazaquin, ^18^F-Pyridaben, and ^18^F-Chromone Analogues

Yu et al. designed and synthesized 4-(2-(4-(4-[^18^F]fluorobutyl) phenyl)ethoxy) quinazoline (^18^F-RP1003), 2-tert-butyl-4-chloro-5-[4-(4-[^18^F]fluoro-butyl)-benzyloxy]-2H-pyridazin-3-one (^18^F-RP1004), and 2-(4-(4-[^18^F]fluoro-butyl)-benzylsulfanyl)-3-methyl-chromen-4-one (^18^F-RP1005) to explore three regular MC-I inhibitor structural classes and examine MC-I inhibitory activity of fenazaquin [59], pyridaben [60], and chromone [61]. The obtained results were identified for optimal probes. Cardiac imaging with ^18^F-RP1003, ^18^F-RP1004, and ^18^F-RP1005 agents in rats and rabbits allowed visualization of the heart with minimal lung interference and rapid liver activity clearance. Moreover, ^18^F-RP1004 PET tracer imaging showed distinct detection of the perfusion-deficit area associated with left coronary artery ligation in rats and marked liver activity washout in nonhuman primates [62]. This result was consistent with previously reported findings using radiotracer rotenone analogues. It is advised that preliminary data should be provided first in healthy subjects for myocardial PET imaging rotenone analogue tracers.

#### 3.1.4. Other ^18^F-Pyridaben Analogues

Recently, researchers used rapid screening tools to evaluate ^18^F-labeled pyridaben analogue tracers and yielded valuable findings. For example, ^18^F-labeled pyridaben analogues were labeled on alkyl side chain rather than directly on the pyridazinone moiety [63]. Mou et al. evaluated the biodistribution and metabolic stability of 2-tert-butyl-5-[2-(2-^18^F-fluroethoxy) ethoxy-benzyloxy]-4-chloro-2H-pyridazin-3-one (^18^F-FP2OP) in mice and verified the results with autoradiography ex vivo. The data from high-yield and radiochemical purity studies (>98%) and biodistribution studies showed that ^18^F-FP2OP PET has a significantly high heart uptake; the imaging showed a clear outline of the myocardium in a healthy Chinese miniswine [64]. This demonstrated the potential value of ^18^F-FP2OP as a myocardial imaging tracer. Another study prompted that 2-tertbutyl-4-chloro-5-(4-(2-(2-(2-^18^F-fluoroethoxy) ethoxy) ethoxy)) benzyloxy-2H-pyridazin-3-one (^18^F-FP3OP) had a better metabolic stability and faster clearance in myocardium compared with ^18^F-FP2OP. However, 2-tertbutyl-4-chloro-5-(4-(2-^18^F-fluoro-ethoxy)) benzyloxy-2H-pyridazin-3-one (^18^F-FP1OP) is worth developing as a new PET MPI tracer owing to its superiority over ^18^F-FP3OP [65]. The instability of ^18^F-FP2OP in water limits its range of applications. These two lipophilic and neutral agents of ^18^F-FP1OP and ^18^F-FP3OP were successfully prepared with high radiochemical yield (~50%) and high purity (>98%). Fenazaquin might have a similar high affinity for the target of rotenone [46]. A clubbed quinazoline and ^18^F-labeled 4-fluorobutyl analogue, designed by Purohit et al., showed a high and rapid heart uptake, fast liver clearance, and low blood uptake in the rat.

Thus, an outstanding MC-I tracer may have several properties: (a) quick myocardial uptake after injection; (b) longer retention in the myocardium; (c) good-quality imaging; (d) uniform radioactive distribution in the ventricular wall; (e) fast radioactivity clearance outside of the heart, such as blood pool, lung, and liver; and (f) no requirement of onsite accelerator production. However, how ^18^F-flurpiridaz meets all the aforementioned requirements in the clinical setting compared with other new MC-I inhibitor tracers who have a hard time getting into the clinic remains to be evaluated. This may be attributed to its better biological characteristics or high sensitivity, but potentially lagging specificity for the detection of CAD [66].

### 3.2. ^18^F-Labeled Voltage Sensor as PET Agents

Mitochondria play a fundamental role in energy metabolism, apoptosis, and oxidative stress [67] in the myocardium, and the mitochondrial inner membrane maintains a transmembrane gradient of ions and integrity of the mitochondrion itself. Measuring the mitochondrial potential shows the development in MBF and perfusion detection and evaluation [68]. Lipophilic cations utilize the mitochondrial transmembrane potential across the cell membrane by passive diffusion and then rapidly accumulate in the myocardium. Hence, ^18^F-labeled lipophilic cation radiotracers are useful for facilitating a clearer appreciation of the myocardial function analysis of a wide range of metabolic dysfunction to the earlier apoptosis-related mitochondrial dysfunction. In these ^18^F-labeled voltage sensor tracers, the active triphenyl phosphonium (TP) group is widely studied. The TP molecule shows electropositive potential and could easily penetrate into the mitochondrial inner membrane, which is a very electronegative organelle [69]. Therefore, TP molecules were commonly used as a labeling radiotracer for apoptosis imaging through detecting mitochondrial membrane potential loss.

#### 3.2.1. ^18^F-Fluorobenzyl Triphenyl Phosphonium

In the mitochondrial or intrinsic signaling pathway for apoptosis, the inner mitochondrial transmembrane potential (ΔΨ*m*) [70] is usually lost, and the mitochondrial release of cytochrome c eventually triggers the activation of caspase. In fact, the release of the apoptosis-inducing factor is dependent on the disruption of ΔΨ*m* early in the apoptotic pathway [71]. Madar et al. first characterized the ability of the novel PET voltage sensor ^18^F-fluorobenzyl triphenyl phosphonium (^18^F-FBnTP) and found a novel radionuclide for PET cardiac imaging in the dog [72, 73], with rapid kinetics, uniform myocardial distribution, and favorable organ biodistribution in vitro and in vivo [74]. Further research yielded that the ischemic region by ^18^F-FBnTP remained stable for at least 45 min and matched the histologically defined ischemic area. This lack of significant redistribution suggests a sufficient time window for future clinical protocols with a tracer injection remote from the scanner, such as in a stress testing laboratory after transient coronary occlusion [75]. Therefore, in a clinical trial practice, ^18^F-FBnTP PET imaging might be one of the most used techniques for myocardium apoptosis.

#### 3.2.2. ^18^F-Fluoropentyl-Triphenyl Phosphonium

The TP cations play a crucial role in detecting the mitochondrial voltage sensor in the outer membrane of myocardial imaging. ^18^F-Fluoropentyl-triphenyl phosphonium (^18^F-FPTP) allows a high throughput and a wide distribution of PET myocardial imaging [76]. 4-[^18^F]-Fluorophenyl-triphenyl phosphonium ion also showed rapid blood clearance and high levels of accumulation in the heart at 30 min. The radioactivity uptake in the heart was 1.64%, 1.51%, and 1.57% ID/g at 5, 30, and 60 min in rats, respectively [77]. More original TP cation tracers should be synthesized and evaluated for healthy subjects and patients with cardiac diseases.

#### 3.2.3. 4-^18^F-Tetraphenyl Phosphonium

TP is useful for measuring ΔΨ*m* in vitro. Although no gold standard method exists for measuring myocardial ΔΨ*m*, measuring ΔΨ*m* has a potential role in assessing heart pathophysiology and therapy as well as myocardial viability. Gurm et al. reported that 4-^18^F-tetraphenyl phosphonium (^18^F-TPP), as a blood flow tracer, was useful in vivo in the PET imaging measurement of ΔΨ*m* for assessing the relative [78], but not absolute MBF.

#### 3.2.4. ^18^F-Labeled (2-(2-Fluoroethoxy)ethyl)tris(4-methoxyphenyl) Phosphonium

Another ^18^F-labeled phosphonium cation agent has been developed due to the technical limitations of SPECT imaging. An ^18^F-labeled phosphonium cation was added to the alkyl group, which increased the hydrophobicity, liposolubility, and hydrophobic interaction between the TP cation and the lipid core. Kim et al. showed that the radiochemical purity was 98% with a 10%–20% yield. The cellular uptake assay showed the preferential uptake of ^18^F-labeled (2-(2-fluoroethoxy)ethyl)tris(4-methoxyphenyl) phosphonium (^18^F-FETMP) in cardiomyocytes, and their biodistribution showed preferential accumulation in the myocardium using small-animal PET imaging studies in mice and rats. The results suggested that ^18^F-FETMP would be a promising candidate for myocardial imaging [79]. Interestingly, the biodistribution properties and preferential uptake of ^18^F-labeled (2-(2-fluoroethoxy) ethyl) triphenyl phosphonium cations in cardiomyocytes were similar to those of ^18^F-FETMP cations in living rats [80]. Moreover, ^18^F-labeled (6-fluorohexyl) triphenyl phosphonium cations showed a low radiochemical yield (15%−20%) [81] and hence require an improvement in the extraction technology in the future. Further evaluation of novel phosphonium cations needs to be carried out in healthy subjects and patients.

#### 3.2.5. ^18^F-4-(Fluoromethyl)benzyl Triphenyl Phosphonium and ^18^F-(3-(Fluoromethyl)benzyl)trisphenyl Phosphonium

TP cations always accumulate in the mitochondria of the heart in response to the negative inner transmembrane potentials. Recently, Zhang's group reported ^18^F-4-(fluoromethyl)benzyl triphenyl phosphonium (^18^F-FMBTP) and ^18^F-(3-(fluoromethyl)benzyl)trisphenyl phosphonium (^18^F-mFMBTP) [82] as potential MPI PET agents, which were obtained in a high radiolabeling yield (~50%) with a good radiochemical purity (>99%) in less than 60 min. Most importantly, these new TP cations were synthesized by the one-pot-labeling procedure and showed high initial radioactivity accumulation in the heart with a rapid nontarget tissue clearance.

#### 3.2.6. ^18^F-Fluorodihydrorotenone B

^18^F-labeled probes for PET imaging have become important tools for directly measuring the ΔΨ*m* [83]. Heinrich et al. developed these fluorescent dyes for MPI. They explored the biodistribution and stability of ^18^F-labeled rhodamine B, also called 3-fluoropropyl ester of rhodamine B, and found lipophilic cation accumulation in the mitochondria in proportion to the mitochondrial membrane potential [84]. The tracer concentration 1 h after injection in the liver was initially greater than that in the heart but decreased over time, which were approximately equal. Throughout this period, the concentration of the tracer in the myocardium remained stable without washout [63]. These findings suggested that the prosthetic group [85], partly due to the diethylene glycol ester, was superior in terms of in vitro stability and pharmacokinetics than ethyl ester for labeled rhodamine B.

### 3.3. FAO as PET Agents

Fatty acid metabolism is a major pathway of energy production in regular perfusion myocardium. In the myocardium, the long-chain fatty acids must be attached to a hitch called coenzyme A (CoA) by enzymes called synthetases in the mitochondria. Therefore, the fatty acid has been renamed as acyl-CoA. *β*-Oxidation of fatty acids mainly occurs within the mitochondria. Products of *β*-oxidation are preferentially channeled to the tricarboxylic acid cycle, away from mitochondrial efflux, via carnitine palmitoyltransferase, which is an enzyme that catalyzes the rate-limiting step in mitochondrial FAO [86]. Hypoxia could prevent long-chain fatty acid-induced accumulation of messenger-RNA encoding muscle carnitine palmitoyltransferase I [87]. The understanding of the rates of myocardial uptake and fatty acid metabolism has led to the development of mitochondrial-targeted fatty acid tracers.

#### 3.3.1. 14(R, S)-^18^F-6-Thia-heptadecanoic Acid

^18^F-Fluorothia-6-heptadecanoic acid (^18^F-FTHA) is a PET tracer with an uptake rate proportional to the rate of myocardial free fatty acid utilization. The results indicated that the metabolic trapping of ^18^F-FTHA in the myocardium occurred subsequent to its entry into the mitochondrion [88]. Noninvasive assessment of myocardial FAO with PET and the development of an appropriate long-chain fatty acid tracer might provide a research and diagnostic tool for evaluating FAO in humans.

#### 3.3.2. 16-^18^F-Fluoro-4-thia-palmitate

Degrado et al. developed 16-^18^F-fluoro-4-thia-palmitate (^18^F-FTP) and showed that moving the sulfur heteroatom to the carbon at position 4 permitted retention of the metabolic trapping character of the tracer [89]. Moreover, they demonstrated that ^18^F-FTP provides high ratios of myocardium activity to the background activities of blood and the lung. Furthermore, the inhibition of mitochondrial *β*-oxidation by oxygen deprivation led to a substantial decrease in the ^18^F-FTP lumped constant (LC) values [90], which is introduced by analogy to the LC used for the estimation of the glucose metabolic rate with deoxyglucose analog radiotracers quantitatively. Developing a novel probe for the myocardial mitochondrial function is particularly necessary for patients with ischemic heart disease.

#### 3.3.3. 18-^18^F-Fluoro-4-thia-oleate

A study on novel 4-thia oleate analogue, 18-^18^F-fluoro-4-thia-oleate (^18^F-FTO), evaluated the difference in biodistribution between fasted and fed rats and found that the livers of fed rats showed a 29% lower uptake. Clearance from the liver was seen between 30 and 120 min in fasted animals, whereas the heart uptake was maintained at a minimal level using small-animal PET/CT. Moreover, a higher heart-to-liver radioactivity concentration ratio was seen with ^18^F-FTO, and the blood-pool data was not corrected for the spillover effect from the myocardium [91]. These results indicated that the PET images of ^18^F-FTO accumulation in the rat myocardium were clearly superior to those of ^18^F-FTP. The ^18^F-FTO PET imaging would show better radio characteristics for myocardium in humans.

## 4. Summary and Outlook

The uptake of ^99m^Tc tracers depends on the mitochondrial membrane integrity of the cell and is seldom influenced by metabolic effects as long as the integrity of the cell membrane is not damaged, even when myocardial cell ischemia, hypoxia, or another condition exists. In this study, ^99m^Tc tracers could be used as reliable indicators of myocardial activity by myocardium stunning. Using ^99m^Tc SPECT for evaluating myocardial viability might underestimate the viability of hibernating myocardium [92, 93]. In contrast, the properties of ^18^F, including active myocardium uptake and redistribution potential, make it a physiological myocardial tracer.

Although PET imaging has a higher spatial resolution than SPECT with high values of sensitivity [94, 95] and allows quantifying the basal and hyperemic regional MBF from metabolism to apoptosis [96], the single PET modality for a variety of clinical applications is not enough. MPI with a hybrid CT device is considerably more expensive and can obtain same-setting measurement of the coronary artery calcium score [97]. However, one obstacle to the wider use of PET/CT is the cost of producing radiopharmaceuticals used for PET imaging in developing countries, compared with SPECT/CT imaging. PET/magnetic resonance imaging (MRI) or SPECT/CT modalities have many advantages in cardiac sarcoidosis or myocardial viability imaging and should be considered for younger patients, as they involve less exposure to radiation [98]. PET/CT or PET/MRI imaging is predicted to extend application from the characterization of atherosclerotic plaques to the efficiency of stem cell therapies with the emergence of novel mitochondrial-targeted tracers [99].

The excellent mitochondrial-targeted myocardial tracers with less liver uptake, higher first-pass extraction, and improved redistribution profile are welcome [28]. Moreover, tracers through a suitable alteration of the molecular properties such as lipophilicity, volume, and charge are needed. ^18^F-TPP was synthesized through direct nucleophilic substitution of no-carrier-added ^18^F-fluoride with the precursor 4-nitrophenyl-triphenyl phosphonium [77], whereas ^18^F-labeled fluoroalkyl triphenyl phosphonium salts (^18^F-FATPs) were synthesized through two-step simple nucleophilic substitution reactions [100]. For both ^18^F-TPP and ^18^F-FATPs, the average radiochemical yield was 10%–30% and the specific activity was >6 TBq/*μ*mol at the end of the synthesis after purification. The total synthesis time was 60 min, and the radiochemical purity was greater than 95%. However, no explicit proof was found regarding one tracer or modality being superior to the other. Physicians involved in nuclear cardiology should review the relative advantages and disadvantages of all ^18^F-labeled myocardial tracers before choosing one for their healthy subjects or patients. Despite ^18^F-FBnTP, ^18^F-FPTP, and ^18^F-FETMP having synthetic low efficiency, it is expected that a number of novel ^18^F-labeled tracers would emerge, with better pharmacokinetic properties of MC-I inhibitor after structural modification.

Overall, the mitochondrial-targeted myocardial molecular imaging of the cardiac processes from myocardial metabolism and apoptosis to ventricular remodeling might be helpful in clinical practices in the future. Each of the available agents has unique advantages and disadvantages that should be considered to ensure its optimal application. The ^99m^Tc-N-DBODC5 and ^18^F-flurpiridaz are commonly used in the clinic for myocardial imaging.

## Figures and Tables

**Table 1:** Mitochondria-targeted molecular imaging agents for myocardium in cardiac function.

Radiotracers	Modality	Location/target	Assessment	In preclinic	In clinic	QL & QT
^99m^Tc-tetrofosmin	SPECT	Inner membrane	MBF		Yes	QL
^99m^Tc-MIBI	SPECT	Inner membrane	MBF		Yes	QL
^99m^Tc-TMEOP	SPECT	Inner membrane	MBF	Yes		QL
^99m^Tc-N-MPO	SPECT	Inner membrane	MBF	Yes		QL
^99m^Tc-N-DBODC5	SPECT	Inner membrane	MBF		Yes	QL
^123^I-BMIPP	SPECT	Fatty acid oxidation	Metabolic		Yes	QL
^123^I-CMICE-013	SPECT	Complex I	MC-I receptor	Yes		QT
^123^I-ZIROT	SPECT	Complex I	MC-I receptor	Yes		QT
^18^F-BCPP-EF	PET	Complex I	MC-I receptor	Yes		QT
^18^F-FDHR	PET	Complex I	MBF	Yes		QT
^18^F-FP2OP	PET	Complex I	MC-I receptor	Yes		QT
^18^F-FP1OP	PET	Complex I	MC-I receptor	Yes		QT
^18^F-FP3OP	PET	Complex I	MC-I receptor	Yes		QT
^18^F-Flurpiridaz	PET	Complex I	MC-I receptor		Yes	QT
^18^F-RP1003	PET	Complex I	MC-I receptor	Yes		QT
^18^F-RP1004	PET	Complex I	MC-I receptor	Yes		QT
^18^F-RP1005	PET	Complex I	MC-I receptor	Yes		QT
^18^F-FBnTP	PET	Inner membrane	MBF/perfusion	Yes		QL
^18^F-FETM	PET	Inner membrane	MBF/perfusion	Yes		QL
^18^F-FERhB	PET	Inner membrane	MBF/perfusion	Yes		QL
^18^F-FMBTP	PET	Inner membrane	MBF/perfusion	Yes		QL
^18^F-mFMBTP	PET	Inner membrane	MBF/perfusion	Yes		QL
^18^F-TPP	PET	Inner membrane	MBF/perfusion	Yes		QL
^18^F-FPTP	PET	Inner membrane	MBF/perfusion	Yes		QL
^18^F-FTPP	PET	Outer membrane	MBF/perfusion	Yes		QL
^18^F-FTHA	PET	Fatty acid oxidation	Metabolic	Yes		QL
^18^F-FTP	PET	Fatty acid oxidation	Metabolic	Yes		QL
^18^F-FTO	PET	Fatty acid oxidation	Metabolic	Yes		QL

MBF, myocardial blood flow; PET, positron emission tomography; SPECT, single photon emission tomography; QL, qualifiable; QT, quantifiable.
